# Structural-functional fingerprinting for abnormalities investigation in glioma patients

**DOI:** 10.21203/rs.3.rs-6590057/v1

**Published:** 2025-05-21

**Authors:** Maria Colpo, Erica Silvestri, Alessandro Salvalaggio, Diego Cecchin, Maurizio Corbetta, Alessandra Bertoldo

## Abstract

Gliomas alter brain function and integrity, but these disruptions are often studied separately. This study utilised a novel approach that integrated functional, structural and microstructural connectivity information to investigate glioma-induced brain network changes and their clinical implications. It focused on the impact of gliomas on key brain networks, with a particular emphasis on the relationship between tumour topology and its effect on homotopic areal-level parcellation. The investigation was grounded in a unique clinical dataset comprising functional and diffusion images of forty-one newly diagnosed glioma patients. Connectivity matrices (functional, structural, and microstructural) were generated using homotopic parcellations and combined into an integration connectivity matrix. A linear regression model compared patient data to pseudo-healthy references. This identified affected regions as those falling in the left tail of the distribution across patients and parcellations. The study revealed that lateralized gliomas affect networks in both hemispheres, with left hemisphere lesions primarily altering homotopic homolateral and contralateral networks in healthy tissues. Abnormalities were more easily detected in regions distant from the lesion using functional connectivity rather than structural measures. The approach highlighted the heterogeneity of functional and structural alterations and emphasised that a comprehensive understanding of glioma abnormalities requires integrating multiple connectivity modalities.

### Introduction

Glioma is the most common group of malignant primary brain tumours in adults[1–3]. This neoplasm is characterised by aggressive invasiveness and infiltration of surrounding brain tissue[4], altering brain function[5] and white matter (WM) integrity[6] through expansion, invasion and intra-tumoral changes[7]. Several studies have examined structural (in WM) and functional (in grey matter (GM)) properties separately, highlighting widespread brain abnormalities.

WM can be studied using diffusion MRI (dMRI), which quantifies molecular diffusion and tissue microstructure and three-dimensional axonal fibers paths. dMRI has been used to inform glioma surgery[8], to characterize major WM tract abnormalities[9,10], and to evaluate microstructure changes[11–13]. However, many studies have focused on perilesional regions rather than widespread brain damage. On the other hand, resting-state functional MRI (rs-fMRI) technique, tracking the hemodynamic fluctuations of the Blood Oxygenation Level Dependent (BOLD) signal, provides information about functional abnormalities induced by glioma[14,15]. To date, most rs-fMRI studies have focused on specific brain networks[16], among them the language network (LANG)[17], the default-mode network (DMN)[18], and the frontoparietal network (FPN)[19], even though the literature in recent years has been proposing new whole-brain approaches[5,20–23]. The next step in the analysis of this data is to integrate dMRI and rs-fMRI at the individual level through a comprehensive whole brain representation, called "integrated connectome"[24–26]. In the context of glioma, understanding the whole brain interplay between structural and functional connectivity could be pivotal for elucidating how alterations in the brain's physical structure influence its dynamic functions, thereby offering crucial insights into the neural mechanisms implicated in glioma-related behaviors, injuries, diseases, and recovery processes.

Structural connectivity (SC), derived from dMRI, quantifies the physical WM projections connecting pairs of brain regions[25]. This measure can be defined by the number of fibers (namely streamlines, NOS) connecting regions[26]. Although widely used, this measure is not without limitations as it is susceptible to various biases and errors inherent in the tractography process[27]. Alternatively, SC can be represented using microstructural diffusion measures averaged along the trajectory of a fibre bundle. For instance, SC can be derived for each pair of connected regions by averaging the Fractional Anisotropy (FA) microstructural values measured along their axonal fibers. Eventually, the two just-described modalities can be joint, weighting the SC matrix characterized by NOS with diffusion microstructure metrics.

Functional connectivity (FC), derived from correlated patterns of synchronised BOLD activity between couples of brain regions[28], represents how functionally integrated areas or networks go beyond physical connections. FC in gliomas enables the identification and localization of functional network alterations[5] and allows the assessment of functional adaptability in relation to tumour resection[20].

Over the past two decades, a great deal of effort has gone into studying the interplay between structural and functional connectivity in the healthy brain[29]. As gliomas disrupt both structural and functional connectivity, understanding their intrinsic relationship is essential for interpreting the broader impact of such pathologies. The innovative aspect of this study lies in introducing a novel approach to integrate structural and functional connectivity and in investigating the added value of an integrated approach over the standard ones, leveraging a clinical dataset with the unique characteristics of including both diffusion and functional data acquired within the same acquisition session. Hence, potentially introducing a clinically valuable biomarker to comprehensively investigate the impact of gliomas on whole-brain networks. Although healthy controls are not available due to clinical constraints, this work highlights the potential of developing a clinically applicable tool to investigate glioma-related structural and functional abnormalities. SC-FC coupling in glioma research remains underexplored, with only a few studies examining the tumour’s impact on both structural and functional brain regions[30,31]. The dynamic interplay between the tumour and the structural and functional aspects of the connectome, as well as the correlation between changes in SC and FC, remains poorly understood[32].

The aim of the present study is to assess whether the quantification of changes in SC-FC coupling: 1) is related to individual stand-alone structural and functional connectivity modalities, 2) contributes to understanding how glioma affects major brain networks, 3) is linked to the topological characteristics of tumours, 4) is able to highlight specific patterns of alteration in homologous brain areas.

While SC provides insights into the brain's physical connections, integrating FC alongside SC offers a more comprehensive understanding of how gliomas disrupt both the structural architecture and functional dynamics of brain networks. Gliomas not only affect the physical connections between brain regions but also alter their functional interactions. By integrating FC, specific functional network alterations induced by gliomas, which may not be evident from SC alone, could be pinpointed. This integrated approach allows to capture the full spectrum of brain network changes associated with glioma progression, providing a more nuanced understanding of the disease pathology and its pattern of progression within the brain. Finally, enriched with insights from integrated SC and FC analyses, novel therapeutic strategies that target the underlying mechanisms driving glioma progression can be devised.

## Results

Glioma-induced changes in SC-FC coupling were investigated using a novel integrated connectivity approach that combines functional, structural and microstructural WM information into a unique concatenated connectivity participant-relevant matrix, the integrated connectivity. Analyses were performed in a clinical context using a unique dataset combining diffusion and functional imaging. To evaluate the impact on homologous regions the Yan homotopic[33] cortical functional atlas (100 parcels, 17 Yeo Networks[34] per hemisphere) was used. Structural connectome matrices were at first quantified according to two different metrics of connectivity: Number of Streamlines (SCnos) and Mean Microstructure Parameter (SCmicro). SCmicro was derived for the following indices from different diffusion microstructure maps: intracellular volume fraction, isotropic volume fraction, orientation dispersion index, FA, mean diffusivity, and mean kurtosis. Moreover, FC represented, throughout Pearson correlation, the temporal similarity between brain regions' dynamic neural activity patterns. Overall, for each patient, connectivity was assessed using four distinct connectivity modalities: SC based on number of streamlines (SCnos), similarity network fusion matrix (SNFmicro) obtained by fusing multiple SCmicro matrices derived from different microstructural indices, FC and integrated connectivity (IC). IC referred to the concatenation, by row, of the FC, SCnos and SNFmicro normalized matrices. For each of the four modalities, to assess the impact of glioma on brain networks, statistically significant differences in connectivity profiles between patients and pseudo-healthy references were examined using a linear regression model. Affected connections/regions were identified as those whose similarity to the expected healthy connectivity profile fell in the left tail of the distribution across regions and patients (i.e., those whose connectivity profile did not fit the expectations). To summarize the glioma-induced alterations at the brain-network and global level we introduced two different metrics: the Network Alteration Degree (NAD), that represents the percentage of altered parcels within the same network, and the Global Disruption (GD) computed as the number of altered networks for each subject. NAD was derived for each patient/connectivity modality, compared across the patients and exploited to investigate the impact of tumour location at the regional level. While GD was used to relate global connectivity changes to clinical information such as overall survival (OS) and tumoral (T), oedematous (O) and lesional (T + O) volumes.

The key finding of the study highlights the effect of glioma on the structural-functional coupling of brain networks, revealing changes close and far from the tumor site. Notably, the disruption is primarily driven by changes in FC within regions that appear structurally intact but are nevertheless affected by the presence of the tumor. Finally, changes in FC, SCnos and SNFmicro were examined in parallel with IC abnormalities to analyse in depth the interplay between integrated and stand-alone connectivities.

The full workflow of the analyses is depicted in Fig. 1.

## • Patients cohort

The patients' cohort comprised forty-one patients (59.5 ± 15 years, 23/18 male/female) affected by glioma at different spatial positions (lesion hemisphere: 22 left hemispheres, 14 right hemispheres, 5 bilateral) and grades (I-IV). Patients were enrolled in the research between July 2017 and April 2021 at the Neurologic Clinic in Padua University Hospital. All the procedures were following the ethical standards of the institutional research committee and with the 1964 Helsinki Declaration plus later amendments. All participants provided written and informed consent per the local Ethic Committee (*Comitato Etico per la Sperimentazione Clinica della Provincia di Padova*, No. 2771P prot:0065859/12). The patients' inclusion criteria are detailed in Supplementary Information 1.1. As reported in Table 1, 26 patients presented wild-type glioblastomas, while 5 patients had the IDH1 mutation. Patients were enrolled between 2017 and 2021 therefore, tumours were classified according to the 2016 WHO classification[35]. Supplementary Table S1 provides the individual demographic and clinical information. Figure 2 shows the frequency maps of the lesions/tumours in the patients' population. More frequently impacted regions predominantly involved association regions with high functional connectivity, such as the left temporal, the right temporal, and the right frontal lobes. The maximum lesion (T + O) overlap value among patients was 22.2%, while the highest tumour (T) overlay value was equal to 19.7%. These results aligned with the expected frequency distribution reported by previous reports[36–38].

Table 1

**Patients' demographics and clinical data.** Demographic summary table with the number of male and female patients, the number of patients with specific tumour histology, the number of patients grouped by the lesion type classification (WHO 2016[35] classification), and the number of patients grouped by the lesion hemisphere position. (y = years, n = number, n.a.= not available, IDH = isocitrate dehydrogenase gene).

| **Age (y)** | **59.5 ± 15** |
|---|---|
| Gender | |
| Female (n) | 18 |
| Male (n) | 23 |
| Tumour Histology | |
| Astrocytoma (n) | 2 |
| Glioblastoma (n) | 29 |
| Glioneuronal and neuronal tumours (n) | 3 |
| Oligodendroglioma (n) | 1 |
| Primary diffuse large B-cell lymphoma of the CNS (n) | 1 |
| Other (n) | 2 |
| n.a (n) | 3 |
| Tumour Grade | |
| Low (n) | 6 |
| High (n) | 32 |
| n.a. (n) | 3 |
| IDH-1 Mutation Status | |
| Wild Type (n) | 26 |
| Mutated (n) | 5 |
| n.a. (n) | 10 |
| Tumour Site | |
| Left (n) | 22 |
| Right (n) | 14 |
| Bilateral (n) | 5 |

### • Examining the relationship between gliomas and integrated and individual connectivity modalities

The study's approach was to emphasize collective structural-functional impairments due to glioma. Additionally, the project established a connection between the IC modality and individual connectivity modalities (FC, SCnos, and SNFmicro). Given the rich literature supporting that the link between SC and FC is complex[29], precluding one-to-one mapping, Fig. 3 illustrates, for each Yeo network[34] across the entire patient cohort, the association between IC alterations and the overlapping single connectivity modality alterations. It should be emphasized that lesion masks, provided by an expert neuroradiologist, encompassed two main lesion tissue types, thus tumoral core (T) and oedematose (O) tissue. Panels A, B and C, correspond to altered networks in overlap with tumour (T), oedema (O) and healthy tissue. It is notable that IC consistently exhibited the highest percentage of alterations, followed by FC, SNFmicro, and SCnos in descending order. Looking at panel B, it is important to mention that networks overlapping with oedema showed a higher frequency of alterations in the right hemisphere. Furthermore, when considering altered networks outside the lesion (T + O) (panel C), SCnos demonstrated no significant impact on the integration connectivity modality.

Supplementary Figs. S1 and S2 display the same representation but group the patients according to the hemisphere lateralization of the lesion. Supplementary Information 1.2 describes the patterns.

### • Unravelling the impact of gliomas on major brain networks

After having investigated the link between integrated and stand-alone connectivities, for each patient, the network-level alteration index (NAD) related to the IC modality was calculated across the 17 Yeo networks[34]. Results were further categorized based on the hemisphere location of the lesions, to supply deeper insights about the impact of gliomas. It should be emphasized that lesion masks, provided by an expert neuroradiologist, encompassed two main lesion tissue types, thus tumoral core (T) and oedematose (O) tissue. Characterizing the pathological tissue type covered by the networks offered a better conception of the glioma's consequences. Networks that overlapped both oedema and tumour were classified according to the tissues they overlapped most.

It could be observed that OS was negatively correlated with the GD index across the patients (Pearson correlation: $r = -0.34, P < 0.05$). Moreover, tumour (T) volume and lesion (T + O) volume exhibited positive correlations with GD measure (Pearson correlation coefficients: $r = 0.51, P < 0.05$ and $r = 0.60, P < 0.05$, respectively (Bonferroni corrected)). The positive correlation became even stronger if considering the relationship between tumour (T) or lesion (T + O) volume and GD linked to networks overlapping with tumour (T) and with lesion (T + O) (Pearson correlation coefficients: $r = 0.74, P < 0.05$ and $r = 0.62, P < 0.05$, $r = 0.64, P < 0.05$ and $r = 0.76, P < 0.05$, respectively (Bonferroni corrected)). Eventually, the Pearson correlation between lesion (T + O) volume and GD derived by networks overlapping with oedema (O) featured a high value (Pearson correlation: $r$ = $0.51$, $P < 0.05$ (Bonferroni corrected)).

Figure 4 displays, in the upper part, the distribution of NAD related to the IC modality for networks that were altered and overlapping with the tumour core (T). Patients with a lesion in either the left or right hemisphere mainly exhibited altered networks within the affected hemisphere. Bilateral patients showed altered networks in both hemispheres. The lower section of the figure represents, in a bar plot, the percentage of altered patients associated with each Yeo network. Examining the graph, it is evident that Right Default-A and Right Salience/Ventral Attention-B networks were the regions most frequently altered among patients, accounting for 29% and 24% respectively. Supplementary Fig. S3 illustrates the overlay between Right Default-A Network and occurrences of lesion(T+O)/tumour(T) (Panel A/B) among patients. It can be appreciated that Right Default-A Network corresponds to areas with higher tumour occupancy.

Examining solely networks affected within the oedematous tissues, Fig. 5 reveals the associated distribution of NAD related to the IC modality. In line with Fig. 4, patients with lesions in either hemisphere predominantly exhibited altered networks within the affected hemisphere. Bilateral patients manifested altered networks in both hemispheres. The lower section of the figure illustrates, through a bar plot, the percentage of impaired patients according to each Yeo network. Upon examining the diagram, it becomes clear that the Right Control-B network was the most frequently altered region among patients (15%). Supplementary Fig. S4 illustrates the overlap between the region of the Right Control-B Network and occurrences of lesion(T + O)/tumour(T) (Panel A/B) across patients.

Given the broader interest in understanding the whole-brain alterations caused by a focal lesion, Fig. 6 presents NAD distributions related to the IC modality of networks out of the lesioned tissues. Left hemisphere patients exhibited alterations overlapping with healthy tissues in both hemispheres. Patients with a lesion on the right hemisphere displayed altered networks primarily in the contralateral hemisphere. Bilateral patients demonstrated abnormalities in both hemispheres. The lower section of the figure depicts, via a bar chart, the proportion of impaired patients associated with each Yeo network. The bar plot emphasizes a lower incidence of alterations in healthy tissues among patients, with Left Limbic-B, Left Default-A, and Right Default-A being the most frequently altered networks (all at 15%). Supplementary Fig. S5 shows the overlap between the just mentioned networks and the lesion(T + O)/tumour(T) (Panel A/B) frequency maps.

It is worth noting that, based on the demographic and clinical data provided in Supplementary Table 1, most patients with an IDH1 mutation did not typically exhibit altered networks overlapping healthy tissues (66,7%). Furthermore, distinguishing patients into low-grade or high-grade tumour diagnosis reveals that subjects with low-grade glioma did not present network alterations without the lesioned area.

Supplementary Figs. S6, S7, and S8 show NAD distributions related to IC, FC, SCnos, and SNFmicro for networks overlapping with tumour (T), oedema (O), and out-of-the-lesioned tissues, respectively. It should be noted that only FC, SCnos, and SNFmicro alterations that overlapped with IC alterations were considered. This choice came from the interest in understanding the weight that the single modalities have in relation to the integrative modality. This approach was guided by the aim of understanding the contribution of each modality in relation to the integrative connectivity framework.

### • Influencing alterations in homotopic parcellation: a glioma perspective

The brain parcellation utilized for this project is characterized by a homotopic correspondence between left and right hemispheres. Considering affected networks overlap with the same tissue type (tumour core (T), oedema (O) and healthy tissue), Fig. 7 illustrates the percentage of IC homotopical altered networks within each patient. Hence, the networks were classified if in overlap with tumour core (T) (panel A), oedema (O) (panel B), or healthy tissues (panel C). When grouping the patients according to the lesioned hemisphere, right and left lesions displayed similar behaviour regarding the pairing of homotopical altered networks within the tumour. In contrast, only left-affected hemisphere patients presented couples of altered homotopic networks outside the lesion. Finally, comparing results with Supplementary Table 1, almost all the patients with altered homotopic networks were classified as high-grade tumours.

The incidence of IC altered pairs of homologous Yeo networks was equal to: 5% for Somatomotor-A, 5% for Dorsal Attention-B, 15% for Salience/Ventral Attention-A, 15% for Salience/Ventral Attention-B, 20% for Limbic-B, 34% for Default-A, and 10% for Default-B across the patient cohort. Specifically, the percentage of occurrences where couples of homologous Yeo networks were IC-based altered and overlapping with the lesion (T + O) varied as follows: 5% for Somatomotor-A, 5% for Salience/Ventral Attention-A, 15% for Salience/Ventral Attention-B, 10% for Limbic-B, 29% for Default-A and 10% for Default-B across the patient cohort.

## Discussion

This study employed a whole-brain connectome approach to comprehensively investigate structure-function connectivity impairments in glioma. The main focus was to integrate FC with diffusion microstructure and structural integrity to further evaluate resulting connectivity impairments.

The main finding highlights the impact of glioma on structural-functional brain network coupling, revealing alterations in tumour-adjacent areas, while the disruption is predominantly dominated by changes in FC in areas that appear healthy but are instead influenced by the tumour.

Homotopic brain parcellation was chosen driven by the necessity to investigate homotopic disruptions caused by the growth of unilateral tumours. Investigating contralateral damage or compensation is of interest because robust synchronous activity between homotopic regions has been shown to underline healthy brain behaviour[15]. Moreover, this approach shifts the focus from isolated SC or FC analysis to their combined insights, revealing how gliomas disrupt connectivity measures and their interplay. Furthermore, by examining contralateral damage or compensation in cases of unilateral tumour sites, we gain valuable insights into the brain's adaptive mechanisms in response to glioma growth. Thus, the integration of multiple modalities provides a more nuanced understanding of the complex interrelationships between structure and function in glioma pathology, guiding future research and clinical interventions.

Confirmative findings of our project are as follows: 1) IC Networks frequently affected are predominantly associated with regions of higher lesion/tumour frequency overlap (as expected); 2) Glioma impacts cerebral functions and structures well beyond the apparent lesion site (as expected).

This project also highlights new findings: 3) Exploring the link between glioma characteristics and topological patterns, patients with lesions on either hemisphere, right or left, present IC alterations of homotopic networks overlaying with the tumour. In contrast, patients with left hemisphere lesions exhibit IC alterations of homotopic networks also in apparently healthy tissues; 4) SC information appears to carry less weight when integrating SC-FC connectivity modalities, such as it offers lower information in SC-FC integration; 5) FC is the most sensitive method to identify areas altered by pathology. Assessing the overall altered areas in regions integrating both functional and structural information, it is evident that as we move from the tumorous zone to the inclusive area encompassing oedema, the significance of functional information becomes increasingly apparent. This mechanism also affects areas of integration that are not adjacent to the pathological zone, where only FC and microstructure, albeit of lesser weight, reveal tissue changes.

The discovery that networks frequently affected by IC are mainly associated with regions with higher overlap frequency, aligns early studies utilizing rs-fMRI[39], dMRI[6,40], and lesion localisation[41]. This result supports the validity of the structure-function integration technique and further sustains the rightness of the developed statistical procedure. Several research groups observed decreased DMN[42–44] or Salience[45] network connectivity, core neurocognitive-related regions, especially in higher-grade gliomas. To date, only one study addressed the structural-functional impairments caused by gliomas, albeit with a focus on specific brain regions (i.e. DMN) and lacking a complete WM microstructure description[30]. Furthermore, our results confirm the oedema role in glioma brain impairments[46].

It should be emphasized that this study highlights an already described behaviour, where SC[6] and FC[47,48] studies have separately reported that glioma induces a widespread impairment of structural and functional properties, also remodelling the neuro-vasculature[15]. The assessment of impairment of the Limbic network, linked to the posterior cingulate cortex and hippocampus, was also suggested by Wey and colleagues[6].

Our results show significant abnormalities described by the integrated approach compared to single-modality analysis. The added value of this project lies in structure-function coupling’s ability to enhance broader brain abnormalities, especially in regions distant from the focal lesion. Therefore, primary brain tumours should be treated as whole-brain disease, affecting the systemic brain rather than localised sites. Healthy tissue regions impaired according to the IC modality may represent new pathways of glioma invasions not yet detected by standard clinical approaches. IC abnormalities may reflect some biological behaviours like tumour progression and invasion or indicate areas of neuroplasticity phenomena.

Homotopic altered networks disruptions highlighted by this research suggest that, despite unilateral nature of the lesions, gliomas may damage connections between mirroring brain regions. Findings of impaired homotopic connectivity in regions outside the lesion are also interesting. The anatomical parcellation applied enhances homotopic correspondence between left and right parcels, derived from a model integrating local and global approaches for estimating areal cortical parcellations[33]. Robust synchronous activity between homotopic regions underlines healthy brain behaviours[36,44]. Daniel et al.[48] explored tumour-induced interhemispheric dysfunction using homotopic connectivity in low- and high-grade gliomas, finding significant associations between homotopic connectivity and tumour severity in high-grade gliomas. Homotopic connectivity disruptions of Somatomotor and Dorsal Attention networks were also significantly associated with OS in high-grade gliomas. Our findings similarly show altered homotopic networks in almost high-grade tumours. Moreover, our project highlights altered homotopic connectivity outside lesioned tissues. This aligns with Hu et al.[49], where frontal glioma patients exhibited reduced homotopic connectivity and resting-state regional function, suggesting that tumours not only cause regional dysfunction but also disrupt long-distance functional connectivity. Correlation analyses also revealed cognitive decline in glioma patients, alongside potential compensatory increases in activity in certain brain regions. In our project, grouping patients according to lesion location and distinguishing networks based on overlap with the lesion highlights homotopic patterns of change outside the lesion for patients with left hemisphere tumours. To our knowledge, this pattern has never been highlighted in the glioma research.

This study unveils the added value of fusing FC, WM integrity and microstructure. Especially in regions distant from the glioma, including functional properties and WM microstructure information provides additional values. Furthermore, IC supplies results not highlighted by individual methods. This might relate to the brain's effort to sustain functions with long-range functional integration in brain tumours. This behaviour may reflect clinical manifestation of chronically progressive tumour growth, preceded by continuous connectome alterations, long before disease manifestation and functional impairments. Structural properties broadly predict functional properties[50,51] thus an integrative approach may furnish complementary information not highlighted by the singles.

Finally, IC alterations may be associated with regions where all individual metrics are affected, or simply with the predominance of alterations in one or more of them. A region altered by IC may indicate that only FC is affected, or rather, that the alteration of even a single metric is so significant as to be integrative highlighted. Thus, our finding suggests that the further from the lesion, the less discriminatory power diffusion metrics demonstrated in detecting alterations.

Regarding the relation with OS, the study provides evidence of a clear relationship between glioma and OS, with a significant anticorrelation between GD index and OS. This result aligns with structural connectome disruption[6] and FC measures significantly correlated with OS[48,52] in glioma.

The project presents some limitations. A major limitation is the absence of a healthy control group matched for age and sex. However, pseudo-healthy references were derived from a unique clinical dataset combining dMRI and rs-fMRI within the same protocol, using a conservative approach to eliminate spurious data. These pseudo-healthy matrices were validated against a healthy dataset, showing comparable graph structures and supporting their validity. The small patient cohort, which includes both high-grade and low-grade glioma based on WHO 2016 classification[35], may limit the statistics obtained. WM tractography, despite state-of-the-art recommendations at the time of the tractogram reconstruction, lacks inter-hemispheric tracts due to SIFT[53] filtering, although the initial high NOS guarantees results reflecting the biology[26,53]. A larger, more homogeneous dataset, a healthy control group, SIFT2[54] or novel diffusion approaches combining microstructure and tractography[55] could improve the described approach. Longitudinal studies are essential to understand IC abnormalities prevalence and translate findings into clinical applications. Finally, since our analysis was primarily driven by SCnos, serving as the basis from which FC and SNFmicro were subsequently selected, it is important to acknowledge that there could be additional alterations beyond this framework. Nevertheless, we deliberately explored this integrated approach.

In summary, gliomas impact was assessed according to an integration connectivity approach, aiming to fuse functional, structural, and WM property information in the assessment of brain tumours abnormalities. This approach highlighted widespread connectivity alterations, providing much more information than single modalities, especially in healthy tissue areas. Eventually, despite the presence of lateralized tumours, altered homologous networks were identified in pathological and healthy regions. Our results confirm the potential of integrated connectivity, which views glioma as a whole-brain disease, to improve clinical outcomes by enhancing commonly used local treatments (i.e. surgery and radiation).

## Methods

### • Participants

Forty-one patients (59.5 ± 15 years, 23/18 male/female) affected by glioma at different spatial positions (lesion hemisphere: 22 left hemispheres, 14 right hemispheres, 5 bilateral) and grades (I-IV) were enrolled in the research between July 2017 and April 2021 at the Neurologic Clinic in Padua University Hospital. All the procedures were following the ethical standards of the institutional research committee and with the 1964 Helsinki Declaration plus later amendments. All participants provided written and informed consent per the local Ethic Committee (*Comitato Etico per la Sperimentazione Clinica della Provincia di Padova*, No. 2771P prot:0065859/12).

As an independent validation dataset, we used data from 200 healthy individuals (59.29 ± 14.28 years, 112/88 M/F) derived from the Human Connectome Project (HCP) in Aging (HCP-A)[56] project. All HCP participants gave full written informed consent prior to the data collection, following Washington University-University of Minnesota (WU-Minn HCP) Consortium ethical guidelines. All procedures were performed in accordance with relevant guidelines and regulations. All the study protocol have been approved by the local Institutional Review Board (IRB) at Washington University in St. Louis.

### • Data acquisition

MRI and PET imaging data were simultaneously acquired at the Nuclear Medicine Unit, Department of Medicine-University Hospital of Padua, on a Siemens Biograph mMR (Siemens Medical Solutions USA, Inc.) PET/MRI scanner equipped with a 16-channels head-neck coil. The MRI protocol included a set of anatomical images, diffusion-weighted images (DWIs) and rs-fMRI images. Details are described in Supplementary Information 2.1.

## • Tumour segmentation and structural pre-processing

The Advanced Normalization Tools (ANTs)[57] (v. 2.0.1) toolbox was used to linearly register all the acquired anatomical images to the T1w image of each patient. After this co-registration process, a mask containing the lesion area was manually delineated through the ITK-SNAP software (http://www.itksnap.org/) by an expert neuroradiologist with more than five years of experience. The mask containing the entire MR-evident lesion was further subdivided into the oedematous tissue (O) and the tumoral core (T), to enable subsequent statistical analyses to account for these different pathological tissues. The tumour core included the contrast-enhancing, non-contrast-enhancing and necrosis areas (when present). Lesion masks were used for pre-processing steps and advanced analyses. Structural processing details can be found in Supplementary Information 2.2.

## • Functional imaging processing

The functional processing of rs-fMRI data followed conventional approaches and is outlined in Supplementary Information 2.3.

## • Diffusion imaging processing, tractogram generation and microstructure estimation dMRI images underwent a diffusion processing described in Supplementary Information 2.4, with the target of generating subject-specific tractograms.

Furthermore, Neurite Orientation Dispersion and Density Imaging (NODDI)[58], Diffusion Tensor Imaging (DTI)[59] and Diffusion Kurtosis Imaging (DKI)[60] models were fitted on dMRI pre-processed data to estimate microstructure maps of the next eight microstructure parameters: intracellular volume fraction, isotropic volume fraction, orientation dispersion index (NODDI model), FA, mean diffusivity (MD) and mean kurtosis (DKI model), FA and MD (DTI model). The microstructure models applied are specified in Supplementary Information 2.4.

## • Structural and functional connectivity computation

The Yan homotopic[33] cortical functional atlas (100 parcels, 17 Yeo Networks[34] per hemisphere) was employed. Overall, for each patient, connectivity was assessed using four distinct connectivity modalities: SC based on NOS (SCnos), similarity network fusion matrix for diffusion microstructure (SNFmicro), FC and integration connectivity (IC). In particular, the first two (SCnos and SNFmicro) are quantitative measures related to the structural connectome, while the functional connectome is measured by FC. IC refers to the concatenation, by row, of the just mentioned matrices after ad hoc normalization. An illustrative representation is displayed in the first row of Fig. 1. Details concerning the generation of the just mentioned connectivity modalities are described below. Procedures to bring parcellation into individual B0 and atlas space are detailed in Supplementary Information 2.5.

## • Structural connectivity matrix generation

For each subject, SC matrix entries represented connection strengths between node pairs. SC matrices were quantified according to two different metrics of connectivity: *Number of Streamlines* (SCnos) and *Mean Microstructure Parameters* (SCmicro).

For SCnos, the process involved superimposing the atlas-based parcellation on the individual whole-brain tractogram and assessing the strength of the connection, in this case calculated as the NOS in the tractogram connecting each pair of parcels. The result was a 200x200 matrix. One-streamline connections were set to zero. Concerning SCmicro, each connectome matrix was weighted by the microstructure, resulting in a 200x200 matrix. Firstly, for each streamline, the microstructure map's value was sampled at each vertex. The mean of these values was then computed to produce a single scalar value of “mean micro” per streamline. Then, as each streamline was linked to nodes coupled within the connectome, the magnitude of the contribution of that streamline to the matrix was multiplied by the mean micro value calculated prior for that streamline; finally, for each connectome edge, the mean value was calculated across the values of “mean micro” that were contributed by all the streamlines assigned to that edge. Again, SCmicro entries for one-streamline connections were set to zero.

It is worth noticing that since we were considering eight microstructure maps, there were eight SCmicro matrices for subject. Thus, after ad hoc standardization (normalization in the range [0-0.99], inverse arctangent and zscore), the eight SCmicro were integrated applying a Similarity Network Fusion (SNF)

approach[61]. The process was applied to construct a fused microstructure matrix called SNFmicro, summing up all the microstructure properties in a 200x200 matrix.

## • Functional Connectivity matrix generation

Regarding FC, the procedure entailed the overlay of the Yan functional parcellation onto the rs-fMRI processed volume. In this case, FC referred to the statistical relationship between rs-fMRI signals of couples of parcels. Pearson correlation was computed between each mean time-series of pair of regions of interest (ROIs), resulting in a 200x200 matrix. Voxels in overlap with the necrotic areas were discarded from the Pearson correlation calculation. Further, parcels retaining less than 20 unaffected voxels were removed. FC cleaning was performed on its Fisher z-transformation (zFC).

## • Integration Connectivity matrix generation

For each subject, the systematic integration of structural and functional modalities was obtained by concatenating, after ad hoc normalization, the single subject FC, SCnos, and SNFmicro matrices, obtaining an IC matrix. In particular, the upper triangular matrices of the three connectivity modalities underwent ad hoc standardization (normalization in the range [0.01–0.99], inverse arctangent and z-score) to reconstruct a normalized square matrix. The three normalized matrices were then concatenated to produce the IC. The purpose was to define an individual structural-functional measure. Each IC matrix size was about 200x600.

## • Statistical Analysis

To explore the added value of an integrated approach compared to the single modes, changes in the connectivity were assessed according to single and integrated connectivity modalities. Connectivity-altered parcels were identified for each connectivity modality, as those parcels significantly differed from a pseudo-healthy template which was derived from the dataset itself. Panel 2 of Fig. 1 illustrates a schematic procedure for deriving parcels' connectivity coherence for a representative connectivity modality for each patient.

All statistics were performed with in-house MATLAB scripts (MATLAB 2023b, The MathWorks, Inc., Natick, MA, USA). The preliminary steps for establishing FC, SCnos, SNFmicro and IC pseudo-healthy matrices are reported below:

- For each subject, all pseudo-healthy entries in the relative SCnos matrix were identified, resulting in a matrix of SCnos cleaned from glioma-related connections (SCnos-cleaned matrix). The criteria for such a selection had essentially two requests to satisfy: 1) for each link, the Yan regions constituting the endpoints of the tract needed to feature an overlapping with the lesion for less than 5% of the total volume of the parcel, and 2) streamlines connecting such endpoints were required not to cross the lesion in any of their points. Moreover, one-streamline connections were set to zero. Then, the SCnos pseudo-healthy matrix was computed as the median across all SCnos-cleaned matrices.

- Concerning SCmicro and zFC, each patient matrix was masked by the corresponding SCnos-cleaned, thus obtaining SCmicro-cleaned and zFC-cleaned matrices. Further, SCmicro pseudo-healthy matrix and zFC pseudo-healthy matrix were computed deriving the median among patients of the respective SCmicro-cleaned and zFC-cleaned matrices. FC pseudo-healthy matrix was obtained from inverse Fisher z-tranformation of zFC pseudo-healthy matrix. In addition, the eight median SCmicro pseudo-healthy matrices were fused according to the previously described SNF procedure[61], to derive the SNFmicro pseudo-healthy matrix. Next, the upper triangular pseudo-healthy matrices of the three connectivity modalities underwent ad hoc standardization (normalization in the range [0.01–0.99], inverse arctangent and z-score) to reconstruct a normalized square matrix. The three normalized pseudo-healthy matrices were then concatenated to produce the pseudo-healthy IC. The pseudo-healthy matrices were utilized to detect significant changes in connectivity coherence to the connectivity of single patients.

It is worth noting that the SCnos pseudo-healthy reference exhibited graph metrics such as global efficiency and modularity that aligned well with expected SC patterns observed in healthy individuals, as demonstrated using an independent dataset for validation. Further details regarding the pseudo-healthy reference and its validation can be found in Supplementary Information 2.6.

To investigate alterations in whole-brain connectivity coherence, differences in FC, SCnos, SNFmicro and IC were analysed between patients and pseudo-healthy-references using linear regression model fit[62]. The general statistical procedure carried on (depicted in Panels 2 and 3 of Fig. 1) was the following:

- Both single-subject and pseudo-healthy connectivity matrices were standardized using an ad hoc procedure, detailed in Supplementary Information 2.7. For each connectivity modality, a linear model was then applied to compare each row of the standardized patient matrix with the corresponding row of the standardized reference matrix. The coefficient of determination $R^2$ value derived from the linear model was used to evaluate the coherence measure between each parcel connectivity profile and its pseudo-reference profile. In Panel 2 of Fig. 1, the connectivity coherence measure for the IC mode is displayed for each parcel and subject on the right side.
- In Panel 3 of Fig. 1, the common strategy for defining altered connectivity coherence measures of parcels for each connectivity modality is depicted. $R^2$ values were labelled as abnormal if belonging to the left lower tail of the $R^2$ distribution across all the parcels/patients. $R^2$ cut-off threshold was chosen equal to 0.25 (e.g., corresponding to a correlation of ± 0.5, i.e. the mean value between the R absolute range). Thus, for each patient, a parcel was defined as potentially altered if the following condition was true:

$$R^2\,(i,j) \leq\ 0.25$$

## Equation (1)

where $(i, j)$ defines the specific parcel *i* and patient *j* examined.

Finally, given the interest in understanding the relationship between structure-function integration and individual modalities in the assessment of whole-brain glioma abnormalities, parcels exhibiting altered FC, SCnos and SNFmicro were masked by those with altered IC and included in further analyses.

Eventually, for each connectivity modality, a vector (i.e., the vector of the impaired parcels) containing all the parcels that were found to be potentially altered was created. Vectors corresponding to the analysed patients were arranged side by side to create a unified matrix with dimensions of 200x41.

## • Network and global degree of alteration

Once the connectivity altered parcels were obtained, a measure of overall alteration for each Yeo network[34] (i.e., node) was derived. In analogy with the graph analysis concept of node degree[63], for each Yeo network, the degree of alteration was evaluated as the percentage of altered parcels within the same network. This value was indicated as Network Alteration Degree (NAD). Supposing each network *n* composed of K parcels, a measure of NAD was defined as:

$$NAD_n = \left(\frac{1}{K}\sum{}_{k=1}^{K} Parcel_{impaired}\left(k\right)\right) \times\ 100$$

## Equation (2)

With this computation, the measure of alteration degree represented, for each network (i.e. node), the amount of alteration degree given by the parcels that belong to the same network.

The computation of Eq. (2) enabled linking the severity of the state of structural-functional abnormalities within each network to its individual connectivity (i.e. FC, SCnos and SNFmicro) alterations.

Panel 4 of Fig. 1 displays the general strategy to define the altered parcels for every connectivity modality.

To highlight the cut-off impact, a sensibility analysis was performed for $R^2$ thresholds in the range [0.15:0.01:0.35]. Details and results are shown in Supplementary Information 2.8 and Supplementary Fig. S9.

Furthermore, the Global Disruption (GD) associated with the IC modality could be assessed by deriving the number of altered networks for each subject. Given N networks, for each subject *j* the GD was computed as:

$$GD_j = \sum\nolimits_{n=1}^{N} (NAD_n > 0)$$

## Equation (3)

Finally, Pearson correlation was used to test the correlations between GD index, overall survival (OS), tumoral (T) volume and lesion (T + O) volume. Results obtained by the comparison of GD values and lesion and tumoral volumes underwent multiple comparison corrections.

## Declarations

#### Supplementary Information

Supplementary information is available at Scientific Reports online.

#### Acknowledgment

Research reported in this publication was supported by the National Institute On Aging of the National Institutes of Health under Award Number U01AG052564 and by funds provided by the McDonnell Center for Systems Neuroscience at Washington University in St. Louis. The HCP-Aging 2.0 Release data used in this report came from DOI: 10.15154/1520707. The research was supported by #NEXTGENERATIONEU (NGEU) and funded by the Ministry of University and Research (MUR), National Recovery and Resilience Plan (NRRP), project MNESYS (PE0000006) - A Multiscale integrated approach to the study of the nervous system in health and disease (DN. 1553 11.10.2022).

#### Author contributions

M.Colpo and AB designed the study and performed the analysis. M.Colpo, AB and ES contributed to the interpretation of the data, and drafting of the article. ES and DC collected the data. M.Colpo, ES, AS, DC, M.Corbetta, and AB reviewed the article and approved its final version.

#### Data and code availability

The oncological data that support this study’s findings are available from the corresponding author, upon reasonable request. The HCP-A 2.0 Release data used in this report came from http://dx.doi.org/10.15154/1520707.

The codes and processed data that support the conclusions of this research work can be accessed via request to the corresponding author.

#### Competing interests

The authors have no competing interests to declare that are relevant to the content of this article.

## References

1. Louis, D. N. *et al.* The 2021 WHO classification of tumors of the central nervous system: A summary. *Neuro Oncol* **23**, 1231–1251 (2021).
2. van den Bent, M. J. *et al.* Primary brain tumours in adults. *The Lancet* vol. 402 1564–1579 Preprint at https://doi.org/10.1016/S0140-6736(23)01054-1 (2023).
3. Lapointe, S., Perry, A. & Butowski, N. A. Primary brain tumours in adults. *The Lancet* vol. 392 432–446 Preprint at https://doi.org/10.1016/S0140-6736(18)30990-5 (2018).
4. Cuddapah, V. A., Robel, S., Watkins, S. & Sontheimer, H. A neurocentric perspective on glioma invasion. *Nature Reviews Neuroscience* vol. 15 455–465 Preprint at https://doi.org/10.1038/nrn3765 (2014).
5. Daniel, A. G. S. *et al.* Functional connectivity within glioblastoma impacts overall survival. *Neuro Oncol* **23**, 412–421 (2021).
6. Wei, Y. *et al.* Structural connectome quantifies tumour invasion and predicts survival in glioblastoma patients. *Brain* **146**, 1714–1727 (2023).
7. D'Souza, S., Hirt, L., Ormond, D. R. & Thompson, J. A. Retrospective analysis of hemispheric structural network change as a function of location and size of glioma. *Brain Commun* **3**, (2021).
8. Henderson, F., Abdullah, K. G., Verma, R. & Brem, S. Tractography and the connectome in neurosurgical treatment of gliomas: The premise, the progress, and the potential. *Neurosurg Focus* **48**, E6 (2020).
9. Mahmoodi, A. L., Landers, M. J. F., Rutten, G. J. M. & Brouwers, H. B. Characterization and Classification of Spatial White Matter Tract Alteration Patterns in Glioma Patients Using Magnetic Resonance Tractography: A Systematic Review and Meta-Analysis. *Cancers* vol. 15 Preprint at https://doi.org/10.3390/cancers15143631 (2023).
10. Friedrich, M. *et al.* Alterations in white matter fiber density associated with structural MRI and metabolic PET lesions following multimodal therapy in glioma patients. *Front Oncol* **12**, (2022).
11. Yan, J. L. *et al.* A Neural Network Approach to Identify the Peritumoral Invasive Areas in Glioblastoma Patients by Using MR Radiomics. *Sci Rep* **10**, (2020).
12. Li, C. *et al.* Characterizing tumor invasiveness of glioblastoma using multiparametric magnetic resonance imaging. *J Neurosurg* **132**, 1465–1472 (2020).
13. Villani, U. *et al.* Diffusion-based microstructure models in brain tumours: Fitting in presence of a model-microstructure mismatch. *Neuroimage Clin* **34**, (2022).
14. Park, J. E., Kim, H. S., Kim, S. J., Kim, J. H. & Shim, W. H. Alteration of long-distance functional connectivity and network topology in patients with supratentorial gliomas. *Neuroradiology* **58**, 311–320 (2016).
15. Hadjiabadi, D. H. *et al.* Brain tumors disrupt the resting-state connectome. *Neuroimage Clin* **18**, 279–289 (2018).

16. Liu, Y. *et al.* Structural and Functional Reorganization Within Cognitive Control Network Associated With Protection of Executive Function in Patients With Unilateral Frontal Gliomas. *Front Oncol* **10**, (2020).
17. Jütten, K. *et al.* Asymmetric tumor-related alterations of network-specific intrinsic functional connectivity in glioma patients. *Hum Brain Mapp* **41**, 4549–4561 (2020).
18. Tordjman, M. *et al.* Functional connectivity of the default mode, dorsal attention and fronto-parietal executive control networks in glial tumor patients. *J Neurooncol* **152**, 347–355 (2021).
19. Jin, L. *et al.* The Functional Reorganization of Language Network Modules in Glioma Patients: New Insights From Resting State fMRI Study. *Front Oncol* **11**, (2021).
20. Hart, M. G., Price, S. J. & Suckling, J. Connectome analysis for pre-operative brain mapping in neurosurgery. *Br J Neurosurg* **30**, 506–517 (2016).
21. Derks, J. *et al.* Connectomic profile and clinical phenotype in newly diagnosed glioma patients. *Neuroimage Clin* **14**, 87–96 (2017).
22. Metwali, H., Raemaekers, M., Ibrahim, T. & Samii, A. Inter-Network Functional Connectivity Changes in Patients With Brain Tumors: A Resting-State Functional Magnetic Resonance Imaging Study. *World Neurosurg* **138**, e66–e71 (2020).
23. Cai, S. *et al.* Hemisphere-Specific Functional Remodeling and Its Relevance to Tumor Malignancy of Cerebral Glioma Based on Resting-State Functional Network Analysis. *Front Neurosci* **14**, (2021).
24. Catani, M., Thiebaut de Schotten, M., Slater, D. & Dell'Acqua, F. Connectomic approaches before the connectome. *Neuroimage* **80**, 2–13 (2013).
25. Hagmann, P. *et al.* MR connectomics: Principles and challenges. *J Neurosci Methods* **194**, 34–45 (2010).
26. Smith, R. E., Tournier, J. D., Calamante, F. & Connelly, A. The effects of SIFT on the reproducibility and biological accuracy of the structural connectome. *Neuroimage* **104**, 253–265 (2015).
27. Calamante, F. The Seven Deadly Sins of Measuring Brain Structural Connectivity Using Diffusion MRI Streamlines Fibre-Tracking. *Diagnostics* **9**, (2019).
28. Friston, K. J. Functional and Effective Connectivity: A Review. *Brain Connect* **1**, 13–36 (2011).
29. Honey, C. J., Thivierge, J. P. & Sporns, O. Can structure predict function in the human brain? *NeuroImage* vol. 52 766–776 Preprint at https://doi.org/10.1016/j.neuroimage.2010.01.071 (2010).
30. Jütten, K. *et al.* Dissociation of structural and functional connectomic coherence in glioma patients. *Sci Rep* **11**, (2021).
31. Liu, L. *et al.* Outcome prediction for patient with high-grade gliomas from brain functional and structural networks. in *Lecture Notes in Computer Science (including subseries Lecture Notes in Artificial Intelligence and Lecture Notes in Bioinformatics)* vol. 9901 LNCS 26–34 (Springer Verlag, 2016).
32. Meyer-Baese, A. *et al.* Controllability and Robustness of Functional and Structural Connectomic Networks in Glioma Patients. *Cancers (Basel)* **15**, (2023).

33. Yan, X. *et al.* Homotopic local-global parcellation of the human cerebral cortex from resting-state functional connectivity. *Neuroimage* **273**, (2023).
34. Thomas Yeo, B. T. *et al.* The organization of the human cerebral cortex estimated by intrinsic functional connectivity. *J Neurophysiol* **106**, 1125–1165 (2011).
35. Louis, D. N. *et al.* The 2016 World Health Organization Classification of Tumors of the Central Nervous System: a summary. *Acta Neuropathologica* vol. 131 803–820 Preprint at https://doi.org/10.1007/s00401-016-1545-1 (2016).
36. Mandal, A. S., Brem, S. & Suckling, J. Brain network mapping and glioma pathophysiology. *Brain Communications* vol. 5 Preprint at https://doi.org/10.1093/braincomms/fcad040 (2023).
37. Mandal, A. S. *et al.* Lesion covariance networks reveal proposed origins and pathways of diffuse gliomas. *Brain Commun* **3**, (2021).
38. Salvalaggio, A. *et al.* White Matter Tract Density Index Prediction Model of Overall Survival in Glioblastoma. *JAMA Neurol* **80**, 1222 (2023).
39. Moretto, M. *et al.* The dynamic functional connectivity fingerprint of high-grade gliomas. *Sci Rep* **13**, (2023).
40. Silvestri, E. *et al.* Assessment of structural disconnections in gliomas: comparison of indirect and direct approaches. *Brain Struct Funct* **227**, 3109–3120 (2022).
41. Sansone, G. *et al.* Patterns of gray and white matter functional networks involvement in glioblastoma patients: indirect mapping from clinical MRI scans. *Front Neurol* **14**, (2023).
42. Esposito, R. *et al.* Modifications of default-mode network connectivity in patients with cerebral glioma. *PLoS One* **7**, (2012).
43. Harris, R. J. *et al.* Altered functional connectivity of the default mode network in diffuse gliomas measured with pseudo-resting state fMRI. *J Neurooncol* **116**, 373–379 (2014).
44. Maniar, Y. M., Peck, K. K., Jenabi, M., Gene, M. & Holodny, A. I. Functional MRI shows altered deactivation and a corresponding decrease in functional connectivity of the default mode network in patients with gliomas. *American Journal of Neuroradiology* **42**, 1505–1512 (2021).
45. Yang, J. *et al.* Glioma-induced disruption of resting-state functional connectivity and amplitude of low-frequency fluctuations in the salience network. *American Journal of Neuroradiology* **42**, 551–558 (2021).
46. Silvestri, E. *et al.* Widespread cortical functional disconnection in gliomas: an individual network mapping approach. *Brain Commun* **4**, (2022).
47. Stoecklein, V. M. *et al.* Resting-state fMRI detects alterations in whole brain connectivity related to tumor biology in glioma patients. *Neuro Oncol* **22**, 1388–1398 (2020).
48. Daniel, A. G. S. *et al.* Homotopic functional connectivity disruptions in glioma patients are associated with tumor malignancy and overall survival. *Neurooncol Adv* **3**, (2021).
49. Hu, G. *et al.* Altered Static and Dynamic Voxel-mirrored Homotopic Connectivity in Patients with Frontal Glioma. *Neuroscience* **490**, 79–88 (2022).

50. Honey, C. J. *et al.* Predicting human resting-state functional connectivity from structural connectivity. *Proceedings of the National Academy of Sciences* **106**, 2035–2040 (2009).
51. Rosenthal, G. *et al.* Mapping higher-order relations between brain structure and function with embedded vector representations of connectomes. *Nat Commun* **9**, (2018).
52. Sprugnoli, G. *et al.* Tumor BOLD connectivity profile correlates with glioma patients' survival. *Neurooncol Adv* **4**, (2022).
53. Smith, R. E., Tournier, J. D., Calamante, F. & Connelly, A. SIFT: Spherical-deconvolution informed filtering of tractograms. *Neuroimage* **67**, 298–312 (2013).
54. Smith, R. E., Tournier, J. D., Calamante, F. & Connelly, A. SIFT2: Enabling dense quantitative assessment of brain white matter connectivity using streamlines tractography. *Neuroimage* **119**, 338–351 (2015).
55. Daducci, A., Dal Palù, A., Lemkaddem, A. & Thiran, J. P. COMMIT: Convex optimization modeling for microstructure informed tractography. *IEEE Trans Med Imaging* **34**, 246–257 (2015).
56. Harms, M. P. *et al.* Extending the Human Connectome Project across ages: Imaging protocols for the Lifespan Development and Aging projects. *Neuroimage* **183**, 972–984 (2018).
57. Avants, B. B. *et al.* A reproducible evaluation of ANTs similarity metric performance in brain image registration. *Neuroimage* **54**, 2033–2044 (2011).
58. Zhang, H., Schneider, T., Wheeler-Kingshott, C. A. & Alexander, D. C. NODDI: Practical in vivo neurite orientation dispersion and density imaging of the human brain. *Neuroimage* **61**, 1000–1016 (2012).
59. Basser, P. J., Mattiello, J. & LeBihan, D. MR diffusion tensor spectroscopy and imaging. *Biophys J* **66**, 259–267 (1994).
60. Steven, A. J., Zhuo, J. & Melhem, E. R. Diffusion Kurtosis Imaging: An Emerging Technique for Evaluating the Microstructural Environment of the Brain. *American Journal of Roentgenology* **202**, (2013).
61. Hansen, J. Y. *et al.* Integrating multimodal and multiscale connectivity blueprints of the human cerebral cortex in health and disease. *PLoS Biol* **21**, e3002314 (2023).
62. MontGomery, D. C., Peck, E. A. & Vining, G. G. Introduction to Linear Regression Analysis. *Wiley* (2012).
63. Bassett, D. S. & Sporns, O. Network neuroscience. *Nature Neuroscience* vol. 20 353–364 Preprint at https://doi.org/10.1038/nn.4502 (2017).

## Figures

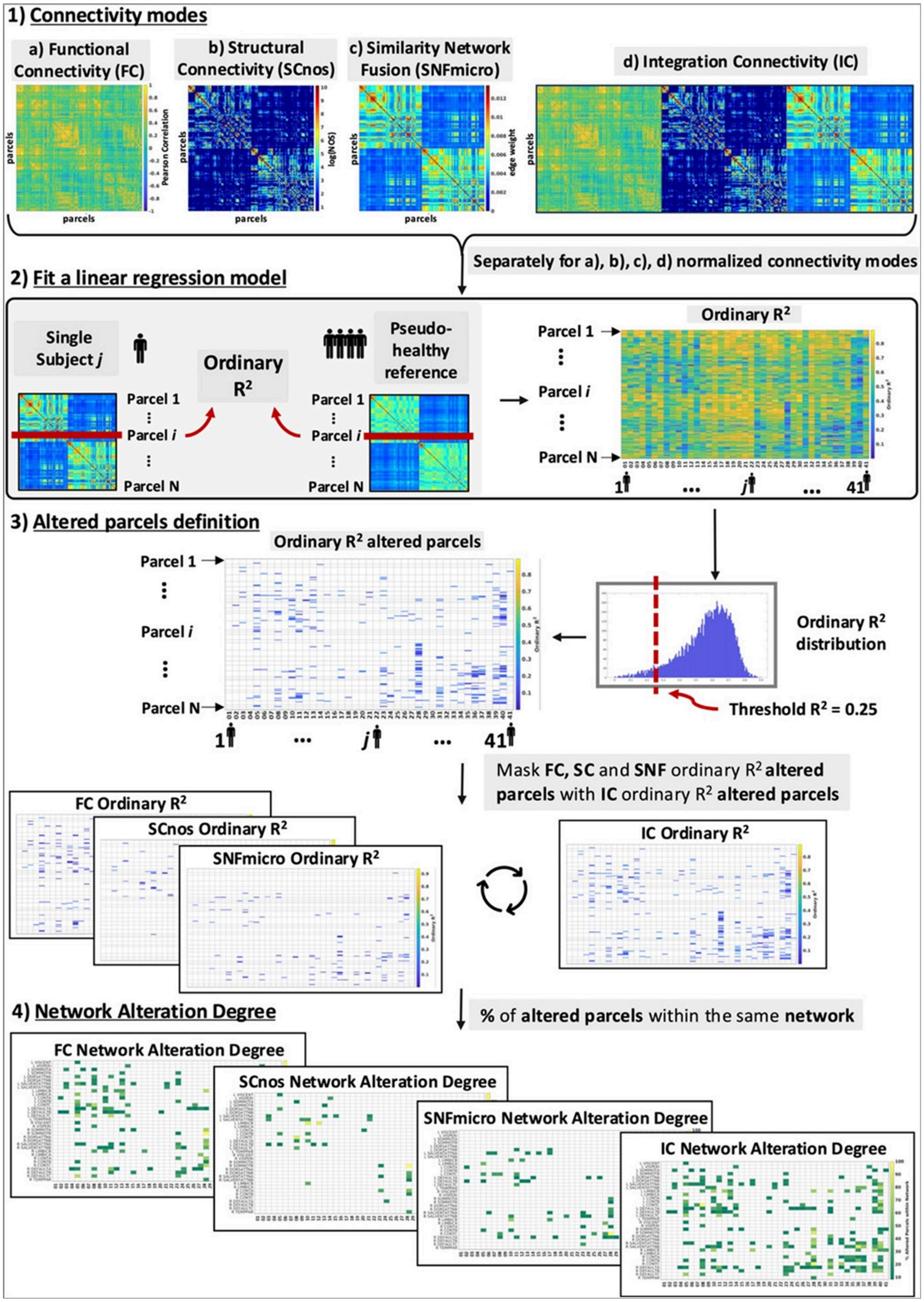

### Figure 1

**Workflow illustration of the analysis procedure for each connectivity mode.** Box 1 outlines the four types of connectivity modes: FC, SCnos, SNFmicro, and IC. Box 2 describes the approach for deriving parcel connectivity coherence for each patent. For each connectivity mode, a linear model is applied between each row of the single patient matrix and the corresponding row in the reference matrix (after ad hoc normalization). Box 3 presents the general strategy for defining altered parcels. Abnormal $R^2$ values are labelled according to the left lower tail of the distribution, utilizing a threshold equal to 0.25. Finally, FC, SCnos and SNFmicro altered parcels are masked by IC altered parcels. Box 4 displays the Network alteration Degree (NAD), representing the percentage of altered parcels within the same network among the patients, for each connectivity mode.

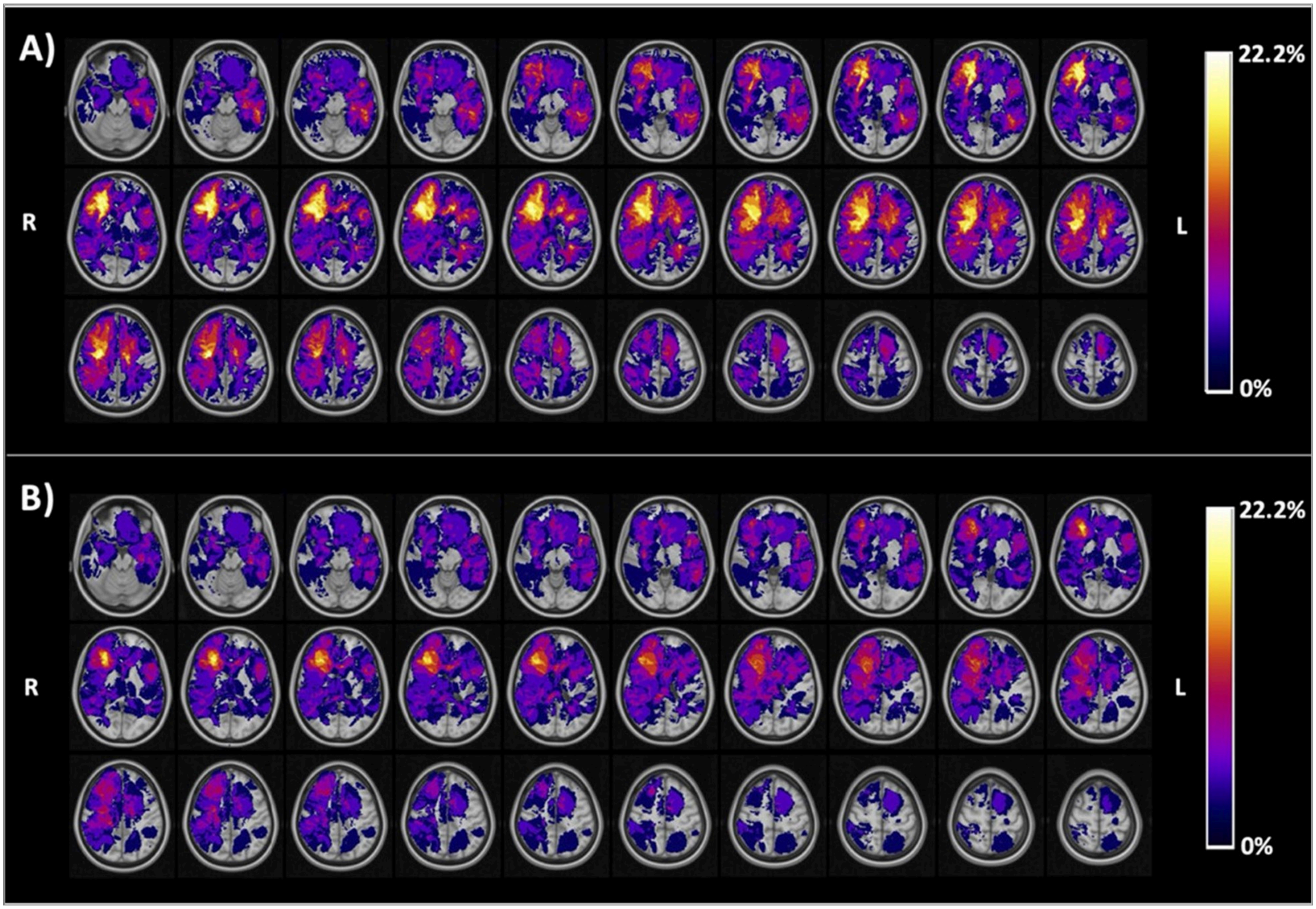

**Figure 2**

**Lesion frequency map across patients' cohort.** (A) Frequency map of tumour lesions including tumour and oedema area (T+O). (B) Frequency map of tumour core (T), excluding oedematous tissues. Maps are over-imposed to the MNI atlas (greyscale). Radiological convention.

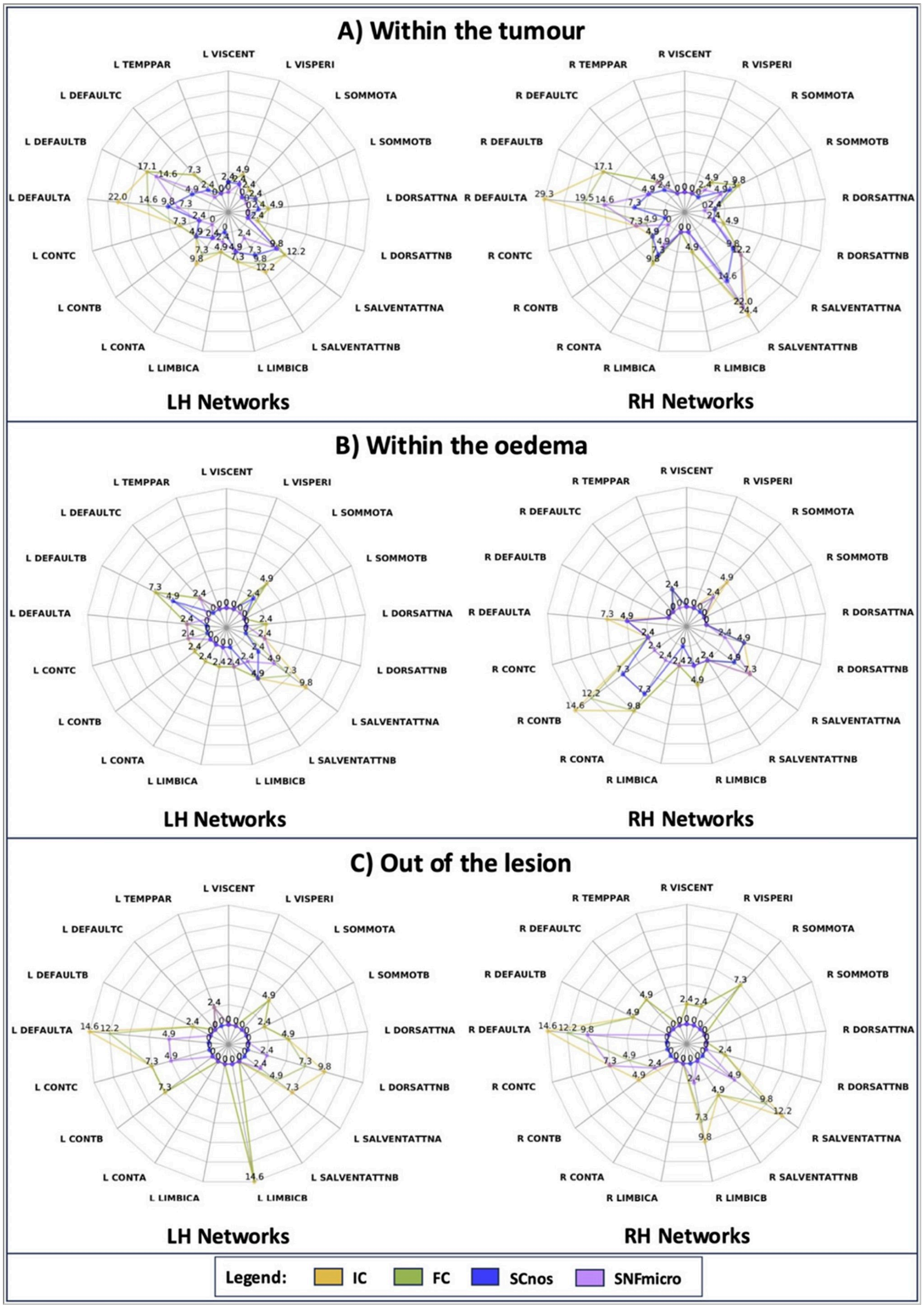

### Figure 3

**Overall percentage of patients with IC, FC, SCnos and SNFmicro altered networks.**In general, the spider plot represents the percentage of patients with altered connectivity associated with each Yeo Network. Networks are grouped according to left and right hemispheres (left and right side of the image). For each pair of spider plots displayed in the same row, the values range is the same. As illustrated in the legend, IC mode in yellow, FC mode in green, SCnos mode in blue, SNFmicro mode in purple. In A), the percentage of patients with altered networks overlapping with the tumour (T) is depicted. In B), the percentage of patients with altered networks in overlap with the oedema (O) is displayed. In C), the percentage of patients with altered networks out of the lesion is shown. VisCent = Visual Central network; VisPeri = Visual Peripheral network; SomMotA = Somatomotor-A network; SomMotB = Somatomotor-B network; DorsAttnA = Dorsal Attention-A network; DorsAttnB = Dorsal Attention-B network; SalVentAttnA = Salience/Ventral Attention-A network; SalVentAttB = Salience/Ventral Attention-B network; LimbicB = Limbic-B network; LimbicA = Limbic-A network; ControlA = Control-A network; ControlB = Control-B network; ControlC = Control-C network; DefaultA = Default-A network; DefaultB = Default-B network; DefaultC = Default-C network; TempPar = Temporal Parietal network.

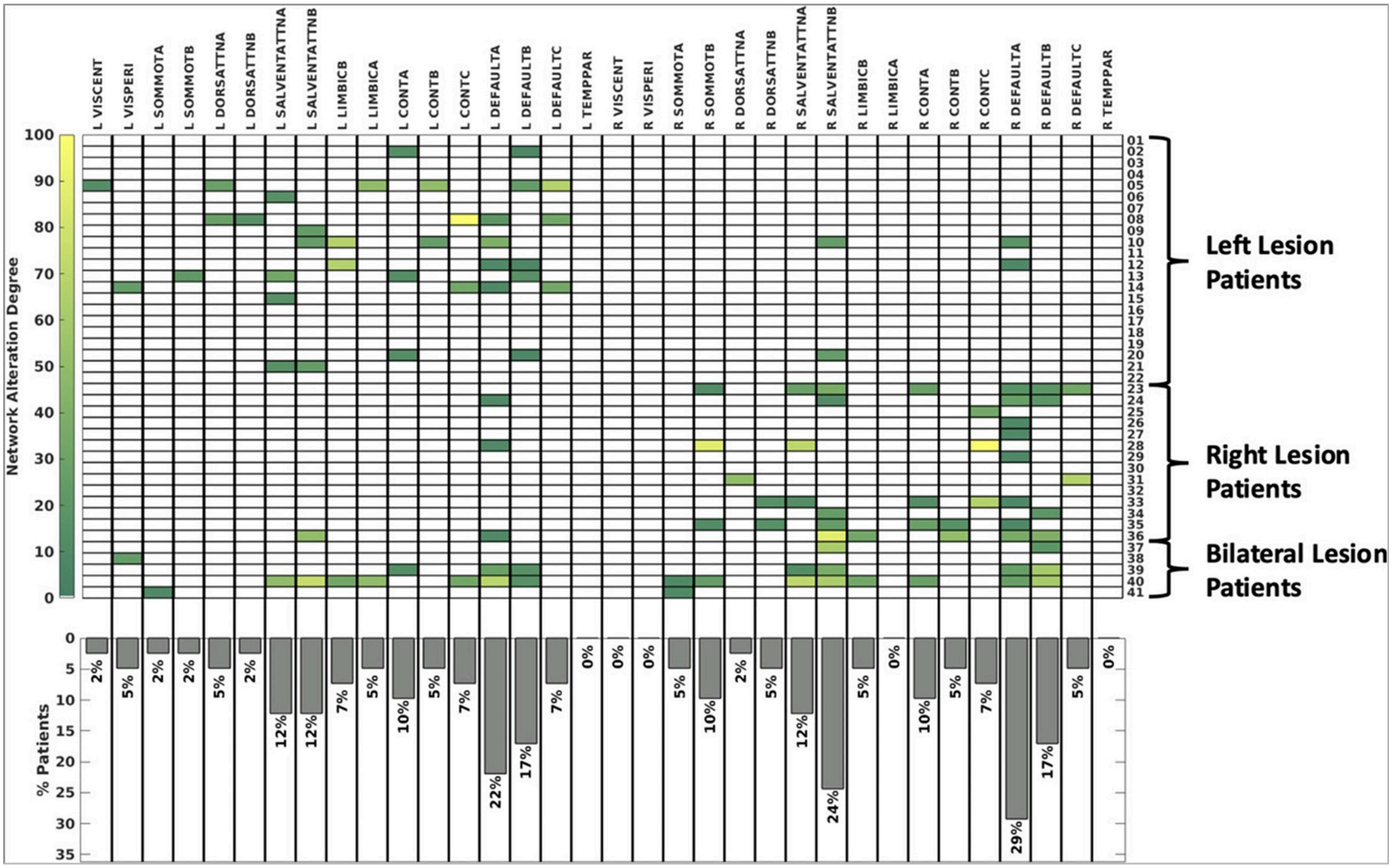

### Figure 4

**Network Alteration Degree (NAD) distribution within the tumour core.** Above: NAD derived from IC for networks overlapping with the tumour core (T). Patients are grouped according to lesion hemisphere position. From the top: left lesion patients, right lesion patients, bilateral lesion patients. Below: Bar chart of the percentage of patients with altered networks. Results are presented for a $R^2$ threshold equal to 0.25. VisCent = Visual Central network; VisPeri = Visual Peripheral network; SomMotA = Somatomotor-A network; SomMotB = Somatomotor-B network; DorsAttnA = Dorsal Attention-A network; DorsAttnB = Dorsal Attention-B network; SalVentAttnA = Salience/Ventral Attention-A network; SalVentAttB = Salience/Ventral Attention-B network; LimbicB = Limbic-B network; LimbicA = Limbic-A network; ControlA = Control-A network; ControlB = Control-B network; ControlC = Control-C network; DefaultA =

Default-A network; DefaultB = Default-B network; DefaultC = Default-C network; TempPar = Temporal Parietal network.

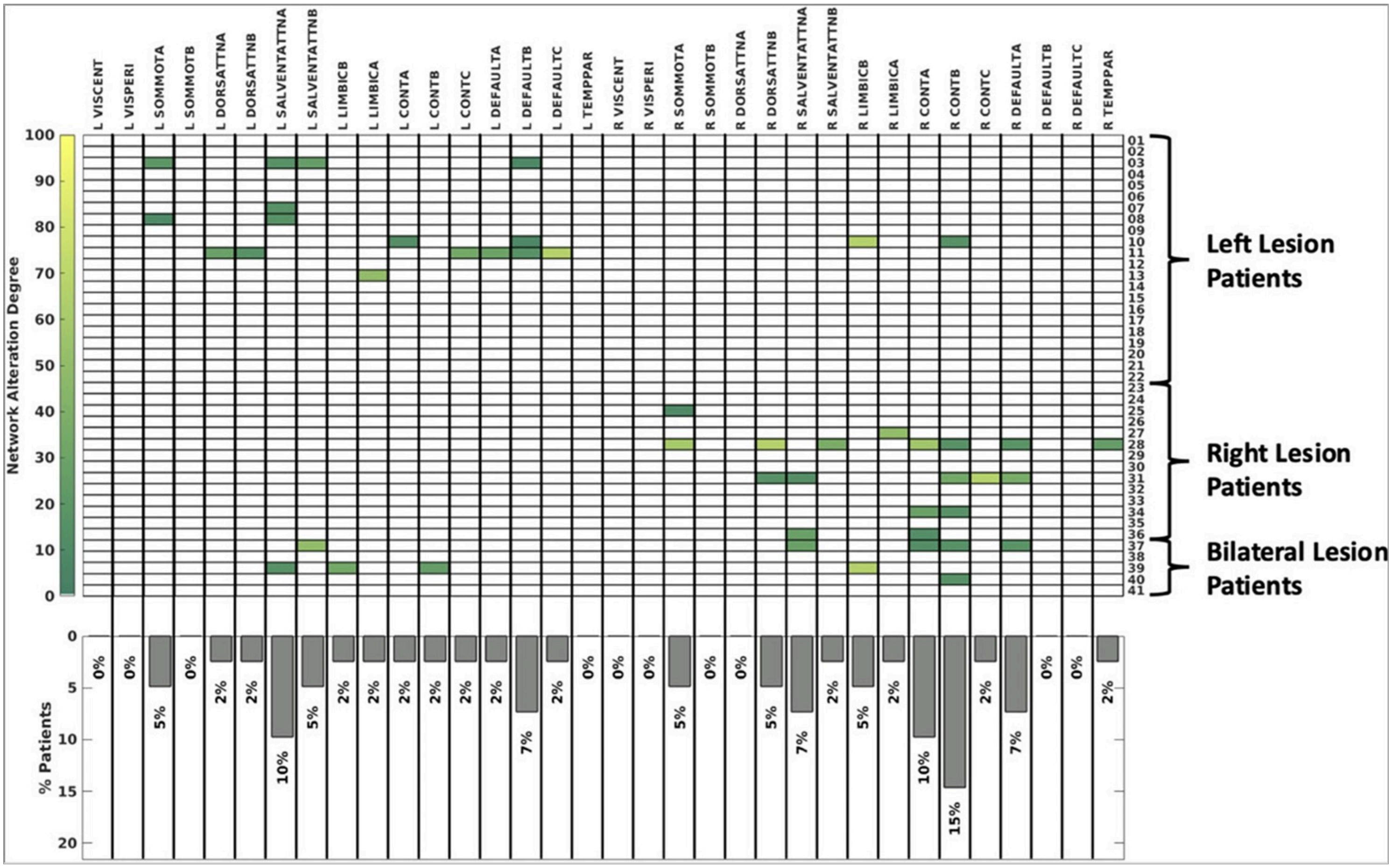

### Figure 5

**Network Alteration Degree (NAD) distribution within oedema.** Above: Network Alteration Degree derived from IC for networks overlapping with the oedematous tissue (O). Patients are grouped according to lesion hemisphere position. From the top: left lesion patients, right lesion patients, bilateral lesion patients. Below: Bar chart of the percentage of patients with altered networks. Results are presented for a $R^2$ threshold equal to 0.25. VisCent = Visual Central network; VisPeri = Visual Peripheral network; SomMotA = Somatomotor-A network; SomMotB = Somatomotor-B network; DorsAttnA = Dorsal Attention-A network; DorsAttnB = Dorsal Attention-B network; SalVentAttnA = Salience/Ventral Attention-A network; SalVentAttB = Salience/Ventral Attention-B network; LimbicB = Limbic-B network; LimbicA = Limbic-A network; ControlA = Control-A network; ControlB = Control-B network; ControlC = Control-C network; DefaultA = Default-A network; DefaultB = Default-B network; DefaultC = Default-C network; TempPar = Temporal Parietal network.

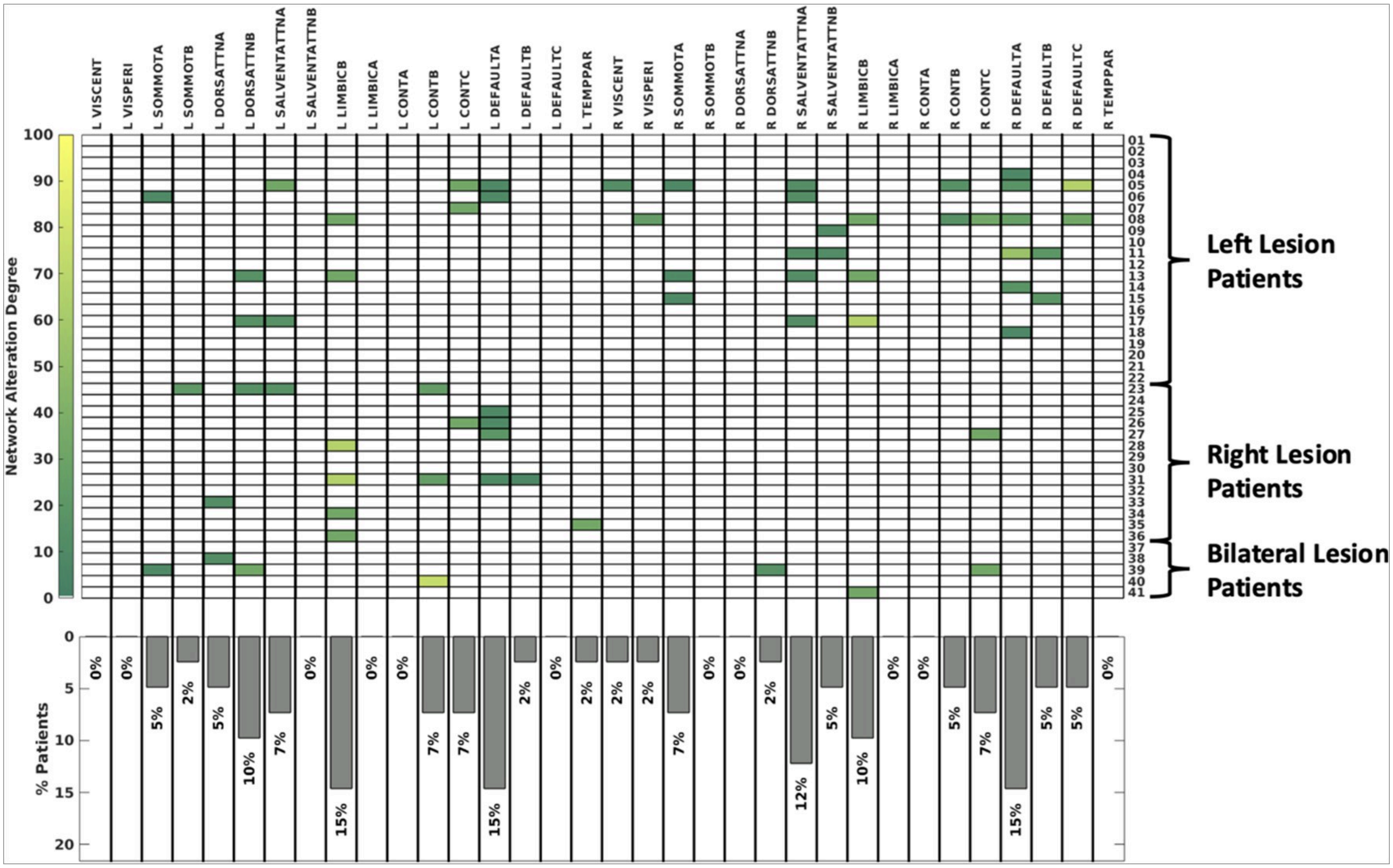

### Figure 6

**Network Alteration Degree (NAD) distribution out of the lesion.** Above: Network Alteration Degree derived from IC for networks without the lesion (tumour core and oedema). Patients are grouped according to lesion hemisphere position. From the top: left lesion patients, right lesion patients, bilateral lesion patients. Below: Bar chart of the percentage of patients with altered networks. Results are presented for a $R^2$ threshold equal to 0.25. VisCent = Visual Central network; VisPeri = Visual Peripheral network; SomMotA = Somatomotor-A network; SomMotB = Somatomotor-B network; DorsAttnA = Dorsal Attention-A network; DorsAttnB = Dorsal Attention-B network; SalVentAttnA = Salience/Ventral Attention-A network; SalVentAttB = Salience/Ventral Attention-B network; LimbicB = Limbic-B network; LimbicA = Limbic-A network; ControlA = Control-A network; ControlB = Control-B network; ControlC = Control-C network; DefaultA = Default-A network; DefaultB = Default-B network; DefaultC = Default-C network; TempPar = Temporal Parietal network.

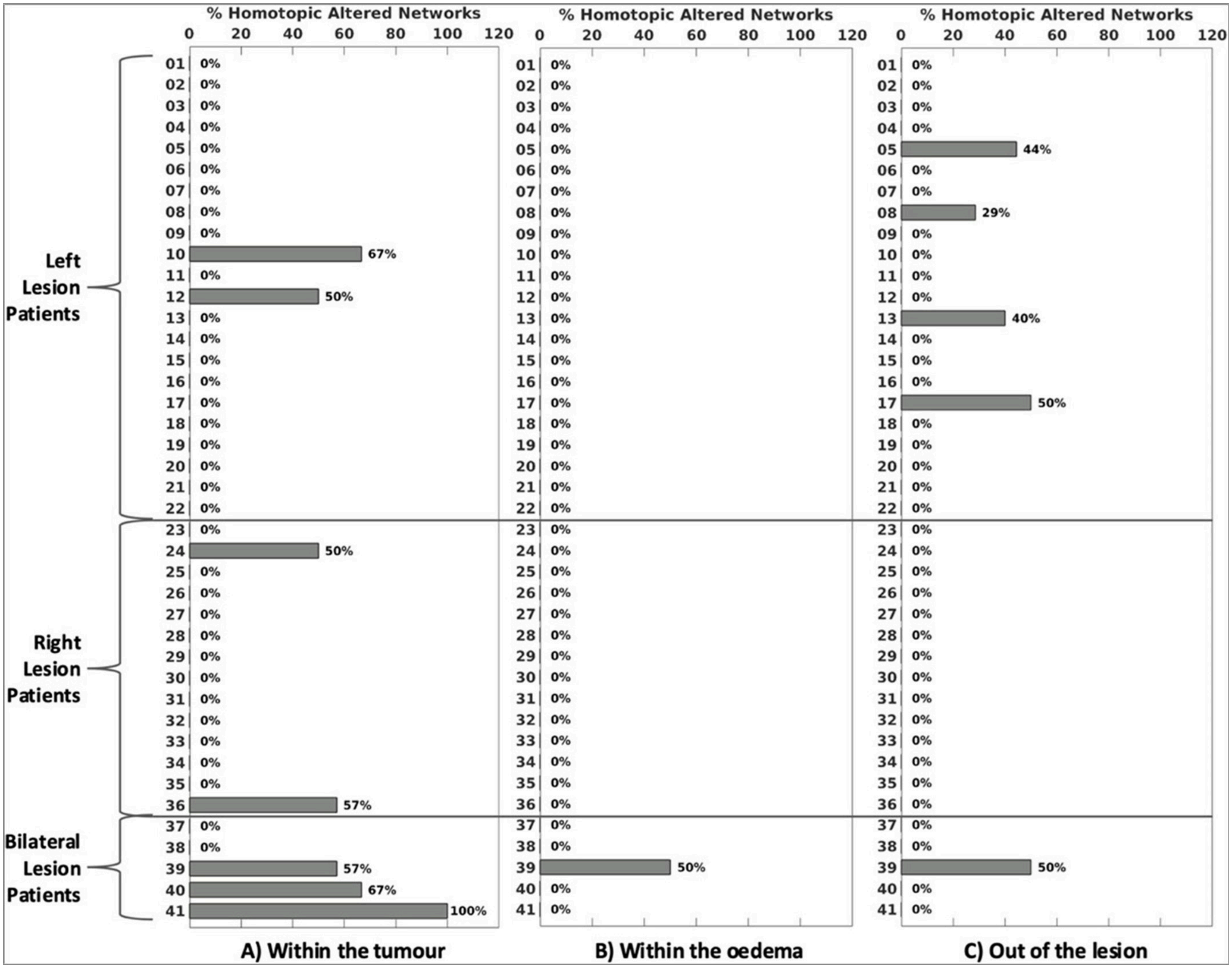

### Figure 7

**Percentage of IC homotopic altered networks.** A) Bar plot of the percentage of homotopic altered networks within the same patients, for networks overlapping with the tumour (T). B) Bar plot of the percentage of homotopic altered networks within the same patients, for networks overlapping with the oedema (O). C) Bar plot of the percentage of homotopic altered networks within the same patients, for networks overlapping with healthy tissues. Patients are grouped according to lesion hemisphere position. From the top: left lesion patients, right lesion patients, bilateral lesion patients.

## Supplementary Files

This is a list of supplementary files associated with this preprint. Click to download.

- SupplementaryMaterial.pdf
